# Assessment of aspirin resistance varies on a temporal basis in patients with ischaemic heart disease

**DOI:** 10.1136/hrt.2008.150631

**Published:** 2009-08-12

**Authors:** A R Muir, M F McMullin, C Patterson, P P McKeown

**Affiliations:** 1 Queen’s University, Belfast, UK; 2 Belfast Health and Social Care Trust, UK

## Abstract

**Objective:**

Laboratory tests including optical platelet aggregometry (OPA), platelet function analyser (PFA-100), and thromboxane B_2_ (TXB_2_) metabolite levels have been used to define aspirin resistance. This study characterised the prevalence of aspirin resistance in patients with ischaemic heart disease (IHD) and investigated the concordance and repeatability of these tests.

**Design, setting and patients:**

Consecutive outpatients with stable IHD were enrolled. They were commenced on 150 mg aspirin daily (day 0) and had platelet function assessment (OPA and PFA-100) and quantitative analysis of serum/urine TXB_2_ at day ⩾7 and then at a second visit approximately 2 weeks later.

**Main outcome measures:**

We assessed the prevalence of aspirin resistance by each method, concordance between methods of measuring response to aspirin and association between time points to assess the predictability of response over time.

**Results:**

172 patients (62.7 (SD 8.7) years, 83.1% male) were recruited. At visits 1 and 2, respectively, 1.7% and 4.7% were aspirin resistant by OPA, whereas 22.1% and 20.3% were aspirin resistant by PFA-100. There were poor associations between PFA-100 and OPA, and between TXB_2_ metabolites and platelet function tests. OPA and PFA-100 results were poorly associated between visits (κ = 0.16 and κ = 0.42, respectively) as were TXB_2_ metabolites, suggesting that aspirin resistance is not predictable over time.

**Conclusions:**

The prevalence of aspirin resistance is dependent on the method of testing. Response varies on a temporal basis, indicating that testing on a single occasion is inadequate to diagnose resistance or guide therapy in a clinical setting.

Antiplatelet therapy, particularly aspirin, forms the cornerstone of treatment for atherosclerotic disease. The Antiplatelet Trialists’ Collaboration reported a 25% reduction in vascular death, myocardial infarction or stroke among high-risk patients treated with aspirin.^1^ Although there have been significant reductions in the morbidity and mortality associated with these disease processes, there continues to be potential for improvement in outcomes.^2^ Atherosclerosis and thrombus formation are complex processes, and the absolute risk of a recurrent event in patients with known disease remains high at approximately 8–18% after 2 years.^3^ Aspirin is classified as a “weak platelet antagonist”^4^ whose effect on inhibition of platelet function is not predictable. Studies have suggested that the antiplatelet effects of aspirin may not be equivalent in all,^5^ leading to the development of the concept of “aspirin resistance”. There is no agreed universal definition of aspirin resistance, making comparison of studies within this field difficult. One use of the term has been to describe patients who continue to experience vascular complications despite aspirin therapy as having “clinical aspirin resistance”,^6^ although other investigators thought this would be better known as “treatment failure”.^7^ Other definitions of aspirin resistance relate to its inability to produce an expected effect on one or more tests of platelet function or on the inhibition of biosynthesis of thromboxane metabolites. These various assessments all attempt to define “biochemical aspirin resistance”; however, the individual defining parameters of each test are not universally agreed. The historical gold standard for assessment of platelet function in acquired platelet function disorders, including following aspirin ingestion, is optical platelet aggregometry (OPA).^8^

The exact prevalence of aspirin resistance or non-response is uncertain owing to the lack of a single, validated method of measurement, leading to a wide range of population estimates from 5.5% to 60%. Given the prevalence of atherosclerotic disease, the potential impact of aspirin resistance on all its clinical consequences is large. Identifying aspirin non-responders and improving their platelet function inhibition by various methods could have a very significant clinical impact.^3^

Our study was designed to characterise the prevalence of aspirin resistance by four different methods (the gold standard optical aggregometry, PFA-100 analyser testing, and both serum and urine thromboxane levels) simultaneously in a cohort of patients with stable coronary artery disease and to assess relations between tests. We also sought to assess the reproducibility of these methods for detection of aspirin resistance by repeating the tests on two separate occasions.

## METHODS

### Patients

In all, 175 patients with stable ischaemic heart disease (IHD) were recruited from October 2004 to June 2006. Three patients failed to return for repeat testing, so results are presented for a total of 172 patients. Stable disease was defined as a confirmed history of a myocardial infarction (MI), percutaneous coronary interventions (PCI) or coronary artery bypass graft (CABG) at least 3 months before enrolment or a history of angiographically proved coronary artery stenosis of ⩾50% stenosis in one or more coronary arteries. Exclusion criteria were a change in symptom complex or confirmed ischaemic event within 3 months of enrolment; ingestion of other antiplatelet agents or non-steroidal anti-inflammatory drugs (NSAIDs) (including cyclo-oxygenase-2 (COX-2) selective anti-inflammatory drugs) within 2 weeks of enrolment; a history of major surgery within 1 week of enrolment; previous significant bleeding; known significant malignant disease or bleeding diathesis; platelet count ⩽150×10^9^/l or haemoglobin count of ⩽8 g/dl. This study complies with the Declaration of Helsinki and was approved by the Queen’s University of Belfast research ethics committee. All patients gave written informed consent.

### Sampling

To maximise uniformity of pharmacokinetics, all patients were prescribed 150 mg of dispersible aspirin daily. Patients attended for tests on two occasions. The first visit took place ⩾7 days following change in aspirin dosage and the second visit occurred approximately 14 days later. Patients continued the same dose and formulation of aspirin between visits. Compliance was assessed by interview at each visit and by telephone between visits. Venesection was performed through a 21-gauge needle and approximately 40 ml of whole blood was collected. All blood samples were processed within 2 hours of collection. A subgroup of patients provided an early morning urine sample taken on the day of assessment. Samples for serum thromboxane B_2_ (TXB_2_) were drawn at the same time as the samples for platelet function testing so that levels of the metabolites could be compared to platelet function results from the same time period.

### Platelet function testing

#### Optical platelet aggregometry

A volume of 20 ml of whole blood was spun at 1000 rpm for 10 minutes to produce platelet-rich plasma (PRP), corrected to a platelet count of approximately 250×10^9^/l using platelet-poor plasma (PPP) as a diluent. PPP was obtained by spinning the remaining cellular suspension following removal of PRP at 3000 rpm for 10 minutes. Aggregation was measured using a Platelet Aggregation Profiler (PAP, BioData Corporation, Horsham, USA) and was recorded as the maximal percentage change in light transmittance from baseline, through corrected PRP, following the addition of various reagents, using PPP as a reference. Aggregometry was measured following the addition of 25 μl of 0.5 mg/ml arachidonic acid (AA), or 25 μl of 10 μM/ml adenosine diphosphate (ADP). Subjects defined as “aspirin resistant” had residual aggregation of ⩾20% following addition of AA and ⩾70% following addition of ADP. These parameters have been used by previous investigators and have been associated with increased risk of adverse events.^8^^–^10 The coefficients of variation for AA and ADP aggregation were 31.2% (at a mean of 62 units) and 6.4% (at a mean of 87 units), respectively.

#### Platelet function analyser

The platelet function analyser (PFA-100, Dade Behring, Marburg, Germany) system provides a quantitative measure of primary, platelet-related haemostasis, by simulating in vitro an artificial vessel wall under shear stress. Whole blood was transferred to the standard cartridges and the time taken to occlude the aperture and prevent blood flow through the membrane coated with collagen and epinephrine (adrenaline) (CEPI) recorded. Subjects with a CEPI ⩽196 seconds despite the ingestion of aspirin were defined as “aspirin non-responders”. This definition has been used by previous investigators and has been associated with increased risk of adverse events.^8^^–^10 The coefficient of variation for PFA-100 was 17.9% (at a mean of 182 units).

#### Serum thromboxane B_2_ and urine 11-dehydro-thromboxane B_2_ measurement

Thromboxane B_2_ metabolites in both serum and urine were measured using enzyme immunoassay kits (Cayman Chemicals, MI, USA). There are no validated levels agreed within the literature above which “aspirin resistance” can be reliably diagnosed; therefore, these measurements were treated as continuous variables in the statistical analysis. Thromboxane metabolites were not normally distributed; hence patients were divided into four equal-sized groups using quartiles of the distribution. The coefficients of variation for serum TXB_2_ and urine TXB_2_ assays were 9.0% (at a mean of 254 units) and 8.6% (at a mean of 134 units), respectively.

### Statistical analysis

Categorical variables are displayed as absolute counts and percentages. In the analysis of associations between alternative measures of resistance, 2-by-2 tables of counts were constructed and the χ^2^ test performed. Thromboxane metabolites were compared between aspirin-resistant and aspirin-sensitive patients using the Mann-Whitney U test. To assess for a relation between serum and urine thromboxane metabolites, results were log transformed and scatterplots constructed. Spearman’s correlation coefficient was used to assess for an association. To assess repeatability between visits, 2-by-2 tables of counts were constructed and the coefficient of agreement, kappa (κ), calculated.^11^ For thromboxane metabolites, scatterplots of log-transformed data were constructed for visit 1 compared to visit 2 and intraclass correlation coefficients (ICC) calculated to assess variation between time points.^12^ All statistics were performed using SPSS for Windows Version 11. Significance was set at the 5% level.

## RESULTS

The cohort of 172 patients who completed the study had a mean age of 62.7 (SD 8.7) years and 143 (83.1%) were male. Patient characteristics are shown in table 1. Using the gold standard method of assessment, optical platelet aggregometry (OPA), the prevalence of aspirin resistance within the cohort was 1.7% and 4.7% at visits 1 and 2, respectively. By PFA-100 38 patients (22.1%) and 35 patients (20.3%) were aspirin non-responders at visits 1 and 2, respectively (table 2).

**Table 1:** Patient characteristics

	Total cohort (n = 172)	Male (n = 143)	Female (n = 29)	Significance
Age (years)	62.7 (8.7)	61.9 (8.6)	66.6 (8.2)	p = 0.006
BMI (kg/m^2^)	30.0 (4.3)	30.0 (4.2)	29.9 (5.2)	p = 0.915
History of smoking*	68.6%	66.2%	80.0%	p = 0.139
History of hypertension†	49.1%	46.9%	60.0%	p = 0.191
History of diabetes‡	21.1%	22.8%	13.3%	p = 0.250
Hypercholesterolaemia¶	76.0%	72.4%	93.3%	P = 0.015
Family history of IHD§	52.0%	51.7%	53.3%	p = 0.872
On β-blocker therapy	88.6%	87.6%	93.3%	p = 0.368
On ACE inhibitor therapy	78.9%	80.7%	70.0%	p = 0.192
On statin therapy	96.0%	95.9%	96.7%	p = 0.838

*History of smoking, smoking within 5 years of ⩾20 pack years. †History of hypertension as per patient records. ‡History of diabetes as per patient records. ¶Hypercholesterolaemia, previous total cholesterol ⩾5.0 mmol/l. §Family history of IHD, first degree relative with confirmed IHD at ⩽60 years.

ACE, angiotensin-converting enzyme; BMI, body mass index; IHD, ischaemic heart disease.

**Table 2:** Prevalence of aspirin resistance in patients with ischaemic heart disease

	Visit 1 (%) (n = 172)	Visit 2 (%) (n = 172)	Coefficient of agreement κ
AA ⩾20%	3 (1.7)	8 (4.7)	0.16
PFA-100	38 (22.1)	35 (20.3)	0.42

AA, arachidonic acid; PFA-100, platelet function analyser.

Median serum TXB_2_ was 90.4 pg/ml (interquartile (IQ) range 43.2–230.2 pg/ml) at visit 1 and 112.0 pg/ml (IQ range 46.5–236.7 pg/ml) at visit 2. Urine samples were collected in a subset of patients (n = 80). Urine 11-dehydro TXB_2_ levels were 395.3 pg/ml (IQ range 232.2–645.5 pg/ml) and 388.8 pg/ml (IQ range 248.5–562.6 pg/ml) at each visit, respectively. There were no significant differences in levels between visits (Wilcoxon signed-rank test p = 0.84 and p = 0.75 for serum and urine, respectively). None of the recorded patient characteristics was independently associated with aspirin resistance by any method but statistical analysis lacked power owing to the small number of patients found to be resistant by the gold standard measurement of OPA.

### Association between measures of resistance

The PFA-100 measurement of aspirin “non-response” was poorly associated with aspirin resistance by OPA. The assessment was limited, however, by the small numbers of patients found to be resistant by the gold standard method. There were no significant associations between the two tests, with the coefficients of agreement κ = 0.02 and 0.22 at visits 1 and 2, respectively (tables 3 and 4). When serum TXB_2_ metabolite levels were compared to the gold standard method of measuring resistance, it was found that they too were poorly associated (table 5). Receiver operating characteristic (ROC) curve analyses showed that it was not possible to use a serum TXB_2_ cut-off point to predict aspirin resistance by OPA (area under the curve (AUC) = 0.50 and 0.46 at visits 1 and 2, respectively). Significant association between PFA-100 and serum TXB_2_ level was observed only at the second visit (table 4). At this visit, aspirin non-responders had a significantly higher level of serum TXB_2_ but there was considerable overlap of values for responders and non-responders. Again, ROC curve analyses indicated that it was not possible to select a cut-off point in serum TXB_2_ level to predict aspirin non-response by PFA-100 (AUC = 0.56 and 0.37 at visits 1 and 2, respectively). There were also no significant associations observed between serum and urine thromboxane metabolites at either visit (data not presented). Urine 11-dehydro TXB_2_ levels were not assessed for an association with aspirin resistance by the gold standard because of small numbers available for the analyses. The urine metabolite was assessed in a subset for an association with aspirin non-response by PFA-100 and this did not reach statistical significance at either visit. This indicated that it was inappropriate to select a urinary level of 11-dehydro TXB_2_, beyond which aspirin non-response could reliably be predicted by PFA-100 (Mann Whitney U test p = 0.99 and 0.36 at visits 1 and 2, respectively).

**Table 3:** Association between aspirin resistance by arachidonic acid aggregation and aspirin non-response by PFA-100 testing in 172 patients (visit 1)

	AA aggregation resistant (%)	AA aggregation sensitive (%)	Total (%)
PFA-100 resistant	1 (33.3)	37 (21.9)	38 (22.1)
PFA-100 sensitive	2 (66.7)	132 (78.1)	134 (77.9)
Total	3 (100.0)	169 (100.0)	172 (100.0)

AA, arachidonic acid; PFA-100, platelet function analyser-100.

Coefficient of agreement between methods, κ = 0.02.

**Table 4:** Association between aspirin resistance by arachidonic acid aggregation and aspirin non-response by PFA-100 testing in 172 patients (visit 2)

	AA aggregation resistant (%)	AA aggregation sensitive (%)	Total (%)
PFA-100 resistant	6 (75.0)	29 (17.7)	35 (20.3)
PFA-100 sensitive	2 (25.0)	135 (82.3)	137 (79.7)
Total	8 (100.0)	164 (100.0)	172 (100.0)

AA, arachidonic acid; PFA-100, platelet function analyser-100.

Coefficient of agreement between methods, κ = 0.22.

**Table 5:** Comparison of serum thromboxane B_2_ levels with resistance by OPA and non-response by PFA-100 resistance cohort (n = 172)

	Resistant by AA aggregation	Sensitive by AA aggregation	Significance
TXB_2_ at visit 1	146.8 pg/ml	89.6 pg/ml	p = 0.99
	(11.0–334.8)	(43.5–230.0)	
	n = 3	n = 169	
TXB_2_ at visit 2	120.9 pg/ml	111.3 pg/ml	p = 0.69
	(28.3–219.3)	(46.6–252.1)	
	n = 8	n = 164	
	**Non-response by PFA-100**	**Sensitive by PFA-100**	**Significance**
TXB_2_ at visit 1	84.2 pg/ml	92.8 pg/ml	p = 0.30
	(37.3–175.6)	(43.5–231.3)	
	n = 38	n = 134	
TXB_2_ at visit 2	194.6 pg/ml	92.9 pg/ml	p = 0.02
	(46.6–544.6)	(46.4–201.1)	
	n = 35	n = 137	

Results displayed as medians and interquartile ranges.

AA, arachidonic acid; PFA-100, platelet function analyser-100; TXB_2_, thromboxane B_2_.

### Associations between visits

Aspirin resistance by the gold standard method of optical platelet aggregometry demonstrated poor association between visits. The coefficient of agreement κ between visits was 0.16 indicating a slight agreement only (table 2). This indicates that aspirin resistance at visit 1 could not reliably predict resistance at visit 2. There was a small improvement in the association between the PFA-100 results between visits, with a coefficient of agreement κ = 0.42, indicating a moderate level of agreement.

In the assessment of the repeatability of serum and urine TXB_2_ metabolites, there were no statistically significant associations between the levels of either metabolite between visits. The intraclass correlation coefficient for the serum metabolite was 0.49 and for urine 0.59, indicating TXB_2_ metabolites varied significantly within patients between visits 1 and 2. This suggests that measurement of either metabolite at visit 1 is unable to accurately predict the level at visit 2.

## DISCUSSION

### Prevalence of aspirin resistance

In our study the prevalence of aspirin resistance by the historical gold standard of OPA was 1.7% and 4.7% at visits 1 and 2, respectively, somewhat lower than that found in other studies. A recent review of the prevalence of aspirin resistance found a mean pooled prevalence (as defined by AA-induced aggregation) of 6% (95% CI 1% to 12%) and was dependent on the dose of agonist used.^13^ In 2001, Gum *et al* assessed patients with stable coronary artery disease and found aspirin resistance by OPA in 5.5%.^8^ In that study, the use of enteric-coated aspirin may account for the difference in prevalence, as the formulation of aspirin ingested affects the amount of aspirin absorbed and, consequently, the degree of platelet function inhibition.^14^ The PFA-100 has been more widely investigated as a method for assessment of platelet response to aspirin because the gold standard method of platelet aggregometry is not suitable for use in the acute setting. The review quoted a mean pooled prevalence of aspirin non-response by PFA-100 of 28.1% (95% CI 22.2% to 33.9%),^13^ with other study results ranging from 20% to 35% in varying atherosclerotic disease processes and with varying doses of aspirin (100–300 mg/day).^15^ ^16^ The lower prevalence in our study may be attributed to improved patient compliance and the use of 150 mg of dispersible aspirin.

### Associations between measures of resistance

The association between platelet function studies is of great importance as prediction of response to aspirin therapy has gained popularity and many clinicians now think this state of abnormal response to aspirin therapy warrants specific therapy. It has been suggested that patients with ACS have higher residual levels of platelet reactivity and that they may benefit “from an alternative or intensified antiplatelet regimen”,^17^ but no single test encompasses all of the distinct and integrated biological events of platelet biology and function.^18^ To date, the only studies to correlate clinical outcome with measures of aspirin resistance have used AA-induced aggregation,^9^ the rapid platelet function analyser^19^ or thromboxane B_2_ metabolites in urine^20^ as their measure of aspirin resistance. In our study, the PFA-100 poorly predicted aspirin resistance by AA-induced OPA, consistent with other studies performed previously.^8^ ^21^ If we assumed that OPA was the gold standard of measuring aspirin resistance, then in this study the PFA-100 had a sensitivity of 67% at visit 1 and 25% at visit 2, with poor specificities of 22% and 18% at each visit, respectively, but the power of this is limited by the small numbers of resistant patients by the “gold standard”. Recently, Faraday *et al* examined the concordance of platelet aggregation, PFA-100, both urinary and serum TXB_2_, fibrinogen, von Willebrand factor and C-reactive protein levels in healthy volunteers.^22^ Of the five patients resistant by aggregometry criteria, four were also resistant by PFA-100. Our study resulted in no significant association between aggregation and PFA-100 at visit 1, but a weak association at visit 2; however, confidence intervals are wide as the study was limited by the low prevalence of aspirin resistance by the gold standard method and it cannot safely be concluded that any association exists between the two tests.

Marcucci *et al* performed PFA-100 assessment in patients undergoing primary percutaneous intervention for acute MI. PFA-100 non-response was a significant independent predictor of the composite endpoint of cardiac death, new MI or target vessel revascularisation,^23^ but assessments were in the acute setting while patients were on other antiplatelet medications; therefore, the association between the PFA-100 results and clinical outcome remains unproved. Some authors suggest that the correct definition of aspirin resistance is a failure to adequately inhibit platelet TXA_2_ production, reflecting the mode of action of the drug.^9^ TXA_2_ is an unstable metabolite; thus, its production is determined by measurement of its stable metabolites in serum and urine. Eikelboom *et al* found that increasing quartile of urinary TXB_2_ was associated with a composite endpoint of cardiovascular death, MI or stroke. This study had many potential confounding variables, such as compliance and measurement in the acute setting, so that elevated TXB_2_ levels may have been caused by a recent event.^24^ Rouvier *et al* correctly suggested that even with significant depression of TXB_2_ metabolites, a normal aggregation pattern can be observed^25^ and the Eikelboom study should not be interpreted as evidence to use therapy targeted at lowering TXB_2_ metabolite levels to reduce future clinical events.

Our study found that serum TXB_2_ metabolite levels were poorly associated with the gold standard method of measuring resistance, which would be in keeping with results in normal volunteers.^22^ Recently authors have dichotomised the continuous variable TXB_2_ by defining cut-off points for aspirin resistance.^26^ When these parameters were applied to our population, there remained no significant association between serum TXB_2_ levels and either aggregation or PFA-100 resistance (data not presented).

### Associations between visits

The response to aspirin is thought to vary on a temporal basis. A few studies have assessed platelet aggregation in patients undergoing prolonged aspirin treatment, the results of which are conflicting.^27^^–^29 Our study is the first one to assess the effect of aspirin on multiple platelet function assessments on a temporal basis. There were no changes in dosage or formulation between visits so that the only variable to be assessed was change in response with time. Three patients at visit 1 were found to be resistant by aggregation and only one of these remained so at the second visit. The numbers involved lack power to allow detailed conclusions to be drawn and further studies are required to assess the relation of aspirin resistance by platelet function testing with time.

A recent meta-analysis reported that 28% of patients were aspirin resistant and were at increased risk of cardiovascular death, acute coronary syndrome, failure of vascular intervention or a new cerebrovascular event compared to sensitive patients.^30^ This suggests that measurement of platelet response to aspirin can predict outcome and should be used to guide therapy. This area, however, remains controversial and has not been conclusively proved to date. Our study found that aspirin resistance measurement varies on a temporal basis, making a single measurement at a single time point inadequate to assess an individual’s platelet response to aspirin.

### Study limitations

This study does not investigate the association between platelet function response and outcome. The correlation of laboratory-defined aspirin resistance with clinical adverse events remains an important issue. We are undertaking ongoing clinical follow-up of this cohort of patients. However, to date, the small number of clinical adverse events precludes current statistical analysis of the data. Statistical analysis for associations between platelet function tests and urine TXB_2_ levels was also limited in this study as urine was collected in a subgroup of patients only. Finally, there are no previously published data on coefficients of variation of the assays used in this study. Some of the coefficients of variation found were higher than would be ideal but this was mainly because of the low level of platelet aggregation found with the AA at OPA in patients treated with aspirin.

## CONCLUSIONS

The phenomenon of aspirin resistance remains a hotly debated topic as there is no universally accepted definition of what aspirin resistance is. This study was undertaken as an independent assessment of the prevalence of aspirin resistance in a cohort of patients with stable IHD in Northern Ireland. We have shown that the prevalence of aspirin resistance is extremely dependent on the measure of platelet function used to assess response and that different methods produce widely varying prevalence rates for resistance that do not correlate well with each other. The prevalence varies on a temporal basis because, at best, only a very weak association was found between results at visits 1 and 2. This suggests that measurement of aspirin resistance at a single time point may not predict the response to therapy over time and, therefore, currently limits the applicability of aspirin resistance testing to guide antiplatelet therapy in a clinical setting.
